# The effect of gonadoliberin analog treatment in precocious puberty on polycystic ovarian syndrome prevalence in adulthood

**DOI:** 10.3389/fendo.2024.1314752

**Published:** 2024-01-24

**Authors:** Dominika Orszulak, Kacper Niziński, Adriana Bil, Aneta Gawlik, Katarzyna Ziora, Agnieszka Drosdzol-Cop

**Affiliations:** ^1^ Department of Gynaecology, Obstetrics and Oncological Gynaecology, Medical University of Silesia in Katowice, Katowice, Poland; ^2^ Department of Gynecology and Obstetrics, Andrzej Frycz Modrzewski Cracow University, Cracow, Poland; ^3^ Department of Pediatrics and Pediatric Endocrinology, Faculty of Medical Sciences in Katowice, Medical University of Silesia, Katowice, Poland; ^4^ Department of Paediatrics, Faculty of Medical Science in Zabrze, Medical University of Silesia, Katowice, Poland

**Keywords:** central precocious puberty, polycystic ovary syndrome, premature thelarche, gonadotropin releasing hormone analogues, early puberty, Rotterdam criteria, gonadotropin releasing hormone analogues treatment

## Abstract

Precocious puberty is diagnosed when pubertal characteristics appear before the age of 8 years in females. The most common form is gonadotropin-dependent, called axial. The primary method of treatment is administration of gonadotrophin-releasing hormone analogues (GnRHa). The aim of the study was to verify hypothesis that GnRHa therapy in the childhood may be of additive risk factor for polycystic ovary syndrome (PCOS) in adulthood.

**Material and Methods:**

The study group consists of 24 women (median age 22 88 years, median BMI 23.5) treated with GnRHa for central precocious puberty in childhood. The control group includes 40 women (median age 23 years, median BMI 25.6) diagnosed with isolated premature thelarche and not using GnRHa in the childhood. Anthropometric measurements, ultrasound examination of minor pelvis and hormonal profile were performed. PCOS diagnosis was based on Rotterdam criteria.

**Results:**

The study confirmed a higher prevalence of PCOS in the study group (50%) than in the control group (10%); p=0.0006. Significant, linear correlation between free testosterone levels and ovarian size was found in the study group (R=0.45 p= 0.03).

**Conclusions:**

GnRHa therapy during childhood may have a potential influence on incidence of PCOS in the adulthood. Therefore, in this group of patients long-term follow-up focused on screening for PCOS would seem beneficial.

## Introduction

Puberty in girls most often begins between 8 and 13 years of age. During puberty many biological changes take place: including the maturation of correct relations in the hypothalamic-pituitary-ovary system, the development of secondary and tertiary sexual characteristics, the pubertal growth spurt. The amplitude of gonadotropin (Gn) pulses increases during puberty. Changes in central nervous system function and the hypothalamo-pituitary axis in particular underpin this process. The increase in amplitude of LH and FSH secretion, causes a greater production of steroid sex hormones, primarily estrogens. The rise in gonadal sex steroid production, following Gn stimulation, is responsible for changes in many tissues, including the genitals, skin, breast, bone etc. (1, 2).

In the vast majority of teenage girls, the first symptom of puberty is the enlargement of the mammary glands - thelarche, followed by an acceleration of the skeletal growth rate, the development of pubic hair - pubarche and the appearance of the first menstruation - menarche (1, 2). The increasing frequency of pulsatile gonadoliberin release is a necessary condition for the initiation of puberty. The age at which girls begin puberty is influenced by environmental, genetic or nutritional factors (3). Precocious puberty (PP) is defined as the development of secondary and tertiary sexual characteristics before the age of 8 in girls. Precocious puberty is classified as central precocious puberty (CPP; dependent on gonadotrophin-releasing hormone; GnRH), peripheral precocious puberty (PPP; independent of GnRH) and benign variants of PP like precocious adrenarche, precocious thelarche, precocious menarche (4, 5).

In the therapy of CPP a long-acting GnRH analogue with 150 times greater effect than endogenous gonadoliberin is used (6). In the first phase after the administration of the preparation, the pituitary secretion of the hormones LH (luteinizing hormone) and FSH (follicle stimulating hormone) is stimulated, and then the threshold of pituitary gland sensitivity increases. Continous GnRHa therapy leads to downregulation of GnRH-pituitary receptors resulting in reduced secretion of gonadotrophins. As a result, in patients with GnRH-dependent precocious puberty, this medicine inhibits the secretion of estradiol and testosterone, which leads to a slower bone maturation (7, 8).

It is believed that female patients with CPP are predisposed to develop features of PCOS in adulthood. However, the observations are unclear and there is much controversy about the risk of PCOS in this group of patients (9, 10).

The aim of the study was to establish the relationship between GnRH analogue therapy in girls with CPP and possible long-term complications – the risk of PCOS in adulthood.

## Materials and methods

The study group included 24 women diagnosed with central precocious puberty in childhood. Primarily, 29 consecutive patients with CPP were recruited as potential participants of the study but 2 patients were lost during follow-up, 2 patients declined participate in the study, 1 patient was excluded due to chronic pharmacotherapy (oral combined hormonal contraception). The remaining 24 (83%) patients formed the well-defined study group. CPP was diagnosed in girls before the age of 8 based on the results of imaging tests, laboratory tests and clinical examination or in girls with early puberty with high acceleration of bone age and low predicted target height.

All patients from the study group were treated with GnRH analogue – the Diphereline preparation (triptorelin acetate). In children weighing less than 20 kg, 1.875 mg of triptorelin was administered intramuscularly every 28 days, while in children weighing more than 20 kg - 3.75 mg of triptorelin every 28 days. Patients from the study group were treated with GnRH analogues at the Department of Pediatrics and Pediatric Endocrinology of the John Paul II Upper Silesian Child Health Center in Katowice.

Inclusion criteria for the study were as following: treatment with GnRH analogues due to CPP, age 18-26 years, women > 2 years after menarche. Exclusion criteria included: chronic pharmacotherapy, systemic diseases (e.g. cardiovascular diseases, diabetes, endocrine diseases, autoimmune diseases), restrictive diet in the last 12 months, PCOS diagnosed in adolescence in patients with no history of CPP, lack of consent.

The control group included 40 patients with a positive history of premature thelarche, without a CPP diagnosis and without treatment with a GnRH analog. All girls were hospitalized in childhood at the Department of Pediatrics and Pediatric Endocrinology of the John Paul II Upper Silesian Child Health Center in Katowice, in order to exclude CPP. During hospitalization, a physical examination was performed. All patients with breast enlargement, which occurs without additional signs of pubertal development and no changes in additional laboratory and imaging tests, were included into the control group.

The other inclusion and exclusion criteria were identical to the study group.

All participants of the research were informed in detail about its purpose and method and gave a written consent to participate in it. The consent of the Bioethical Committee of the Medical University of Silesia in Katowice to conduct the research was obtained (consent no.: KNW/0022/KBI/135/13/14).

In the first stage of the research, all women underwent a medical interview, including:

- basic demographic data: age, place of residence,
- gynecological interview, which assessed, inter alia, the menstrual cycles, symptoms of hyperandrogenism, obstetric interview and maternity plans, family history of PCOS, hyperandrogenism and infertility,
- general history of medical conditions, mental disorders, history of bone fractures in the past.

Then the anthropometric measurements (height, weight, BMI) were obtained.

The severity of hirsutism was assessed according to the Ferriman-Gallwey scale. Hirsutism was diagnosed with a score of 8 or more points, while the biochemical features of hyperandrogenism were determined after the measurements of ovarian and adrenal androgen levels.

All patients qualified for the study underwent an ultrasound examination of the smaller pelvis (in non-virgin women with use of the transvaginal transducer, in virgins with use of the transabdominal transducer – patients with full urinary bladder).

After the anthropometric measurements in the morning (between 8:00 and 9:00), in the fasted state, 16 hours after the last meal, 15 ml of venous blood was drawn from all examined women (in the early follicular phase, between the 2nd and 5th day of the menstrual cycle). Concentrations of: estradiol, FSH, LH, prolactin, Sex Hormone Binding Globulin (SHGB), total testosterone, dehydroepiandrosterone sulfate (DHEA-S), thyroid-stimulating hormone (TSH) in the blood serum were determined by the ECLIA method using Roche reagents, whereas the manual ELISA method and IMMUNIQ reagents were used to assess the concentrations of androstedione, Anti-Müllerian hormone (AMH) and free testosterone in the blood serum.

The diagnosis of polycystic ovary syndrome was based on the ESHRE/ASRM criteria, which were established in Rotterdam in 2003 and revised in 2012. Diagnostic criteria of PCOS in women were: anovulatory menstrual cycles; clinical and/or biochemical symptoms of hyperandrogenism; image of polycystic ovaries in the ultrasound examination (presence of at least 12 follicles with a diameter of 2-9 mm or the volume of the ovary > 10 ml). PCOS was diagnosed if at least 2 out of 3 criteria were met.

In the statistical analysis of the obtained results, the computer applications Excel 2007 and STATISTICA v.12PL were used. Continuous variables are presented as median and 5% and 95% percentile. Continuous variables were compared using Mann Whitney U test. Categorical variables (reported as numbers with percentages) were tested using χ2 statistics with Fisher’s exact. The result of the analysis was considered to be statistically relevant if the obtained significance level p was less than or equal to 0.05.

## Results

### General characteristics of the studied groups

The mean age of the participants from the study group was 22 years (19; 25), while in the control group it was 23 years (18; 26). The difference between the two groups in this parameter was not statistically significant. There were no statistically significant differences between the two groups also in terms of body weight and body mass index (**Table 1**). The age of menarche was lower in the study group (median 11 years, 8;13) than in the control group (median 12 years, 10;16) with p=0.006.

**Table 1:** Basic characteristic of anthropometric data in the study and control groups (median, 5% and 95% percentile are given, respectively)

Statistical parameter	Study group	Control group	The Mann-Whitney U test
Height [cm]	164 (158; 172)	164 (156; 170)	NS
Body weight [kg]	62.0 (50.5; 77.7)	68.5 (50.0; 110.0)	NS
BMI [kg/m^2^]	23.5 (19.8; 28.4)	25.6 (19.1; 39.0)	NS

NS, no statistically significant; BMI, body mass index.

### Comparison of biochemical parameters in the study and control group

In the next stage of the study biochemical parameters were evaluated (**Table 2**). A difference appeared in the FSH level, with higher values in the study group (p=0.0006), while no statistically significant difference was observed in LH concentrations and the derivative of LH/FSH ratio. The study group had lower median levels of total testosterone, androstenedione and free testosterone, as well as free androgen index (FAI), compared to the control group. All these differences were statistically significant.

**Table 2:** Biochemical parameters in the study and control groups (median, 5% and 95% percentile are given).

Parameter	Reference values	Study group	Control group	Mann-Whitney U test
DHEA-S [μg/dL]	65.1-368	250 (127; 589)	300 (174; 421)	NS
FSH [mlU/mL]	3.5-12.5	6.80 (5.36; 8.42)	5.36 (3.42; 8.36)	p=0.0006
SHBG [nmol/L]	26.1-110	54.6 (20.0; 174.5)	51.6 (27.3; 108.3)	NS
Total testosterone [nmol/L]	0.290-1.67	0.357 (0.089; 0.670)	0.547 (0.234; 0.952)	p=0.0002
TSH [μlU/mL]	0.27-4.2	2.70 (0.83; 3.79)	1.90 (0.82; 2.77)	p=0.01
Androstenedione [mg/mL]	0.7-3.1	2.95 (1.83; 4.77)	4.22 (2.36; 6.25)	p=0.0006
Free testosterone [pg/mL]	0.04-4.18	0.84 (0.45; 5.76)	1.69 (0.89; 4.06)	p=0.002
Estradiol [pg/mL]	12.5-166	61.2 (48.9; 91.1)	85.6 (48.8; 163.4)	p=0.002
LH [mlU/mL]	2.4-12.6	8.63 (5.02; 10.81)	7.66 (2.57; 12.14)	NS
Prolactin [μlU/mL]	102-496	339 (124; 721)	442 (229; 489)	p=0.02
AMH [ng/mL]	0-10.6	8.49 (6.02; 16.90)	9.39 (2.53; 10.67)	NS
LH/FSH	Near to 1	1.26 (0.81; 1.62)	1.40 (0.59; 2.23)	NS (p=0.06)
FAI [%]	<5	0.58 (0.10; 3.12)	0.92 (0.35; 2.20)	p=0.0007

NS, no statistically significant; DHEA-S, dehydroepiandrosterone sulfate; FSH, follicle-stimulating hormone; SHBG, Sex Hormone Binding Globulin; TSH, Thyroid-stimulating hormone; LH, Luteinizing hormone; AMH, Anti-Müllerian hormone; FAI, Free Androgen Index.

Another evaluated parameter was TSH, being significantly higher in the study group. There were no significant differences in DHEA-S, SHBG and prolactin levels between groups. There was also no statistically significant difference in AMH levels, but there was a statistically significant difference in the distribution of results in relation to the norm limit. A higher percentage of women with AMH levels above 10 ng/mL was observed in the study group (Fisher’s exact test: p=0.01) (**Table 3**). When analyzing the study and control groups, the difference regarding estradiol levels was noted; this hormone had lower values in the study group.

**Table 3:** Hormonal profile of selected parameters with regard to reference values.

Parameter	Values	Study group	Control group	Fisher’s exact test
		Number (% of group)	Number (% of group)	
Total testosterone	Below normal	9 (37.5%)	4 (10%)	p=0.01
	Normal	15 (62.5%)	36 (90%)	
	Above normal	0	0	
Androstenedione	Below normal	13 (54.2%)	7 (17.5%)	p=0.003
	Normal	11 (45.8%)	33 (82.5%)	
	Above normal	0	0	
Free testosterone	Below normal	0	0	p=0.05
	Normal	21 (87.5%)	40 (100%)	
	Above normal	3 (12.5%)	0	
AMH	Below normal	0	0	p=0.01
	Normal	16 (66.67%)	37 (92.5%)	
	Above normal	8 (33.3%)	3 (7.5%)	
FAI	Below normal	6 (25%)	2 (5%)	p=0.03
	Normal	18 (75%)	38 (95%)	
	Above normal	0	0	

AMH, Anti-Müllerian hormone; FAI, Free Androgen Index.

In the study group, Ferriman-Gallwey scale was 9.5 (7.2; 18.8) and in the control group 7.0 (0; 14.2) (median, 5% and 95% percentile, respectively); p=0.004. **Table 4** shows the percentage of women who presented features of hyperandrogenism. Consecutively, 87.5% of women in the study group reported complaints of hirsutism (according to Endocrine Society 2018 Guidelines, the diagnosis of hirsutism in Caucasian women of reproductive age is indicated by a score of ≥8 points - modified Ferriman Gallwey scale) and 47.5% in the control group and this constituted a statistically significant difference. Androgenic alopecia occurred in 17.5% of patients in the control group, while none of the woman in the study group reported such a symptom and this was also a statistically significant difference p=0.03. Regarding the occurrence of acne, there was no significant difference, the percentages were 45.8% vs. 45%.

**Table 4:** Symptoms of hyperandrogenism in both groups.

Size	Study group	Control group	Fisher’s exact test
	Number (% of group)	Number (% of group)	
Hirsutism	21 (87.5%)	19 (47.5%)	p=0.001
Acne	7 (45.8%)	18 (45%)	NS
Alopecia	0	7 (17.5%)	p=0.03

NS, no statistically significant.

The prevalence of polycystic ovary syndrome, diagnosed according to the Rotterdam modified criteria among women in the study and control groups, was calculated. Polycystic ovary morphology (PCOM) was statistically significantly more frequent in women from the study group (54.17% vs 15%; p=0.001). Finally, PCOS was confirmed in 50% of women in the study group and in 10% of the control group, with p=0.0006 (**Table 5**). The presence of PCOS disorders in the family history was more frequent in the study group than in the control group (5/24; 21% vs 0/40; 0%, respectively; p= 0.006).

**Table 5:** Prevalence of PCOM and PCOS in both groups.

Size	Study group	Control group	Fisher’s exact test
	Number (% of group)	Number (% of group)	
Ultrasound image of PCOM	13 (54.2%)	6 (15%)	p=0.001
Diagnosis of PCOS according to modified Rotterdam criteria	12 (50%)	4 (10%)	p=0.0006

PCOM, Polycystic Ovarian Morphology; PCOS, Polycystic Ovary Syndrome.

**Figure 1:**
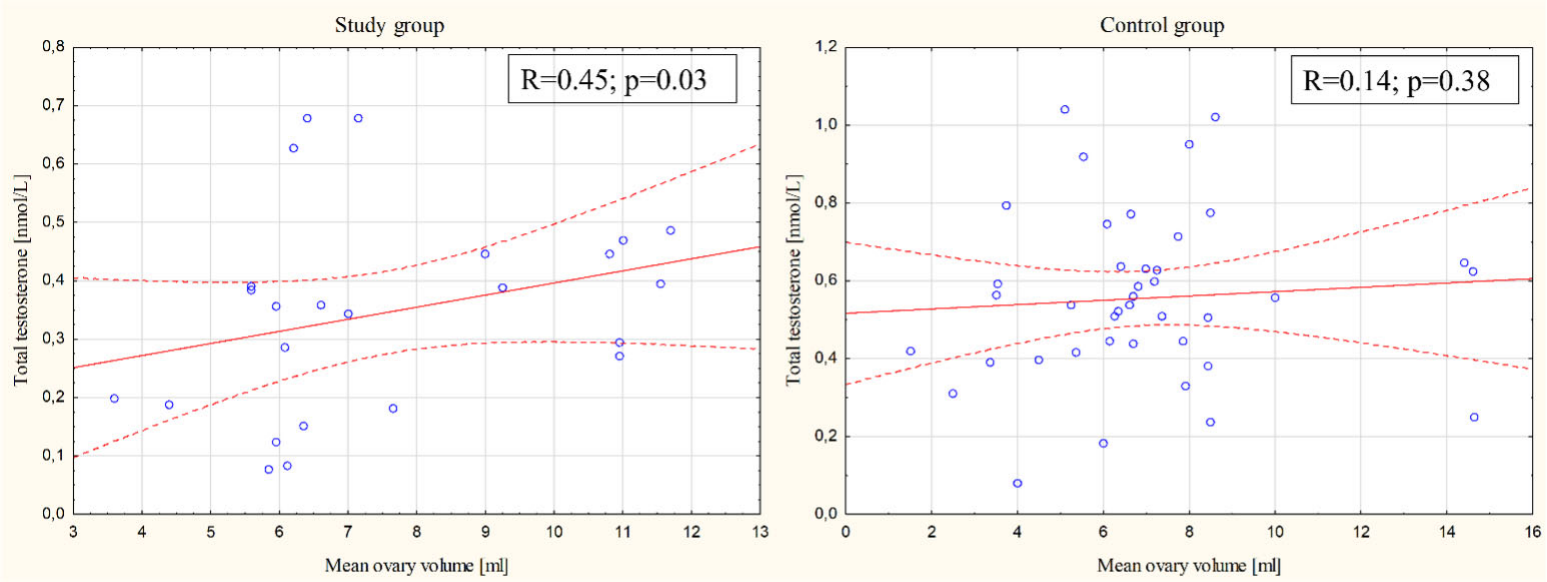
Correlation between total testosterone and mean ovary volume in both groups.

The correlation between the average volume of the ovary and androgen concentrations (total testosterone, free testosterone, and androstenedione) was also investigated. In terms of total testosterone concentration, there was a positive correlation with the volume of the ovary in the study group (Spearman R=0.45; p= 0.03) but not in the control group (Spearman R=0.14; p= 0.38) (**Figure 1**). There were no other statistically significant differences between the study group, the control group and the general population for other evaluated parameters.

## Discussion

Polycystic ovary syndrome is the most common endocrine pathology in females of reproductive age. Based on the high degree of variability between the different diagnostic criteria, there is a unique challenge that exists when determining the prevalence of this syndrome. Due to this reason, the prevalence of PCOS varies between 10% to 13% based on which diagnostic criteria are used: the National Institutes of Health criteria, Rotterdam criteria, or Androgen Excess-PCOS Society criteria (11).

PCOS is characterized by anovulation, hyperandrogenism and polycystic ovaries (12). In this study, we intended to provide novel data concerning the prevalence of PCOS among young women who were treated in childhood with GnRHa due to CPP. The diagnosis of PCOS evaluated in adulthood was based on Rotterdam criteria. The main finding of the study is that the prevalence of polycystic ovary syndrome and polycystic ovarian morphology were higher in the treated group than in the control group (PCOS: 50% vs 10%; p=0.0006 and PCOM: 54.2% vs 15%; p=0.001; respectively). That’s what’s intriguing, in the study group we observed significantly lower androgen levels (total testosterone, free testosterone, androstenedione and FAI) than in the control group (**Table 2**). According to the Rotterdam criteria, hyperandrogenemia or clinical signs of androgenization must be met for the diagnosis of PCOS. However, biochemical hyperandrogenism is only needed if clinical hyperandrogenism is not evident (13). In our study, symptoms of hyperandrogenism as well as ultrasound image of multifollicular ovarian morphology were high in the study group (88% and 54%, respectively). Moreover, the concentration of androgens in women is not directly proportional to the intensity of clinical features and might result from increased sensitivity of androgen receptors (14).

Although there have been several attempts in recent years to evaluate the effects of treatment with GnRH analogues on the developing body and their possible further metabolic and endocrine complications, the results are inhomogeneous and even mutually exclusive. The prevalence of PCOS in CPP patients varies depending on the characteristics of the patients, duration of treatment, follow-up period and differences in PCOS diagnosis standards (15). Due to the low prevalence of precocious puberty in the general population, the study and control groups are usually heterogeneous. In patients with CPP, hormonal (mainly gonadotropin) disorders, hyperandrogenism and PCOM may occur despite the applied GnRHa treatment. It remains unclear whether GnRHa therapy per se modifies the prevalence of PCOS or it results from CPP disease itself. The report by Kim EY et al. (16) from 2015 stated that the prevalence of PCOS among CPP patients was 24%, compared with 2% in the age-matched control group. Therefore, we have made an attempt to correlate the applied treatment with GnRH analogues due to CPP with the occurrence of PCOS.

Some of recent studies reported a high prevalence of polycystic ovary syndrome in CPP patients after GnRHa treatment, but it remains unclear whether the cause is the reproductive mechanism of CPP or GnRHa treatment itself. Faienza et al. (17) published a research paper aimed at investigating the effect of treatment with GnRH analogues on metabolism, skeletal status and the incidence of polycystic ovary syndrome in girls diagnosed with idiopathic central precocious puberty. The incidence of PCOS was higher in the study group concluding that GnRHa therapy is associated with hyperandrogenism, increased risk of insulin resistance and PCOS, but no effect on BMI or changes in lipid profile were found. Among other studies that confirmed an increased risk of PCOS after the use of GnRHa, was the one carried out by Chiavaroli et al. (9) who found that treatment with GnRHa was an independent risk factor for the development of PCOS among subjects diagnosed with CPP.

On the contrary, Heger et al. (10) concluded that there is no effect of GnRH analogues on the reproductive function of examined women and no difference in the incidence of PCOS between the treated and untreated group. Franceschi et al. (18) reported the prevalence rate of PCOS in 46 adult women who were previously diagnosed with CPP and received GnRHa treatment. PCOS was diagnosed according to the Rotterdam and Androgen Excess Society definitions in 32% and 30% of the patients, respectively. That study reported that ovarian hyperandrogenism and polycystic ovarian morphology may occur in CPP regardless of GnRHa treatment. Thornton et al. (19) found no relationship between GnRH treatment and an increased risk of PCOS, as well as other negative health repercussions such as decreased bone mineral density or weight loss. Guaraldi et al. (20) during their observations found that girls treated with analogues for CPP did not have fertility problems, unlike those not treated. Magiakou et al. (21) found that frequency of PCOS incidence in the treated group is 17.2% and 30.8% in the untreated group. Satitpatanapan et al. (22) evaluated 67 women with CPP who had been followed-up for 6-20 years after cessation of GnRHa treatment. Only two of them developed PCOS. The low prevalence of PCOS of 3% suggests that CPP is not a risk factor for PCOS, at least during early adulthood. Finally, meta-analysis of Xaioping Luo et al. (23) included 5475 individuals from 98 studies. Evidence-based comparative studies showed that GnRHa treatment increases the final adult height and decreases the body mass index in girls with idiopathic CPP but does not evidently increase the risk of PCOS.

In our research we have reported a higher incidence of PCOS among CPP patients treated with GnRH analogues. As discussed before, the results remain divergent between researches. Small amount of research on this issue, different criteria for the diagnosis of PCOS and the significant heterogeneity of the groups result with incoherent conclusions. Meta-analysis of Xaioping Luo et al. (23) revealed the overall risk of bias of the eligible studies ranged from critical to moderate and the overall quality of evidence for each outcome ranged from very low to moderate. Especially, the employment of a specific control group is controversial and vague. We have included into control group girls with isolated premature thelarche and not using GnRHa in the childhood. This group does not constitute a model control group according to the principles of conducting clinical trials. The ideal control group should consist of patients with CPP who are untreated. However, in real life it is extremely difficult to find parents refusing the proposed treatment for their child. Parents generally approve proposed GnRHa treatment, hence there are no conditions to create an “ideal” control group. Another potential control groups could be either tall girls with ‘early puberty’ entering into puberty between 8-9 years of age or girls with CPP who enter puberty relatively late (somewhere between 6-8 years of age) who are relatively tall and who present with only moderate advancement of sexual and bone maturation. Those two groups may not require GnRHa treatment but in real life it is difficult to refuse parents who request treatment. The combination of parental expectations, well-intending prescribers, and pressures of the market place can lead to increased utilization of certain drugs. Especially, that young girls were diagnosed at the beginning of 21th century and at that time GnRHa treatment could have been more widely used (due to overmentioned reasons). Even nowadays justification of treatment often relies on assumptions about morbidity (e.g., psychological distress) or undesired outcomes (e.g., reduced adult height) that seem reasonable but, in fact, are not well documented (24). Moreover, the global tendency toward the lowering of the age of onset of puberty observed for several decades might also lead to proclivity of GnRHa treatment (25). Therefore, it is important to emphasize that we must avoid overtreatment, despite an inclination to initiate GnRHa treatment. It is obvious that not all girls really need GnRHa treatment. Recommendations by an international consortium evaluating the use of GnRHa (26) suggest that girls older than 7 years with Tanner stage 2 breast development and early-maturing girls (onset 7-9 years) might undergo an initial observation period of 4–6 months to assess the tempo of pubertal progression before offering treatment. In this group of patients an adequate final height can often probably be attained without the need treatment and may not significantly improve with treatment (especially if at baseline the height is above average). Additionally, their limited advance of secondary sexual characteristics will not cause them major psychological problems. Discussion with the parents remains the crucial point of therapy. Thoughtful, scrupulous presentation of the situation, reassurance of parents and patients, informing them that the benefit of treatment may be uncertain in this age group, is a key aid to lessening psychological distress (26).

Other researchers who were dealing with the problem of the control group either did not include the control group (10) or matched healthy subjects from general population (18). We have decided to create congenial conditions and we have included patients with a similar diagnosis, however, with the awareness that they do not constitute a representative control group.

Moreover, discrepancies in PCOS diagnosis criteria make it difficult to compare different studies with each other. PCOS is clinically a heterogeneous entity, with no single diagnostic test. Diagnostic criteria have evolved from original 1990 National Institute of Health (NIH) criteria. Then in 2003 the ESHRE/ASRM international consensus workshop group met in Rotterdam and set a criteria for adult women. Finally, in 2018 the International Evidence-based Guideline (EBG) for Assessment and Management of PCOS (27) have ratified the Rotterdam criteria which were approved by 38 professional societies and endorsed by the NIH. Researchers usually employ one of the three criteria: Rotterdam criteria; National Institute of Health criteria or Androgen Excess Society&PCOS criteria. Franceschi et al. (18) found similar prevalence of PCOS regardless of the applied criteria (32% had PCOS according to the Rotterdam ones and 30% had PCOS according to the Androgen Excess Society&PCOS criteria). Similarly in our work, the general results would not be affected if we had adopted other criteria. If we used Androgen Excess criteria, in the study group there would be 13 patients with PCOS and in the control group there would 4 patients with PCOS (based on Rotterdam criteria: there are 12 patients with PCOS in the study group and 4 patients with PCOS in the control group).

Due to heterogeneity of symptoms, together with concomitant metabolic and hormonal disorders, it is difficult to determine the etiopathogenesis of PCOS. The cause is complex and includes genetic and epigenetic susceptibility, hypothalamic and ovarian dysfunction, excess androgen exposure, insulin resistance, and adiposity-related mechanisms (13). In our study we have confirmed a possible genetic predisposition, PCOS was more frequent in the family history among women from the study group. Literature data (28) emphasize also the role of immunological factors and oxidative stress in the pathophysiology of PCOS. The risk factors for PCOS also include: gestational diabetes, prematurity, low birth weight, precocious puberty and obesity.

Treatment of GnRHa in CPP is both safe and efficacious. Potential harmful effect is unconfirmed, the adverse effects of GnRHa therapy (allergic reactions, withdrawal bleeding, hot flashes, prolonged QT interval) are rare, and the associations of most reported adverse events with the GnRHa molecule itself are unclear (26). However, due to a potential risk of PCOS, girls after treatment of GnRHa must be strictly and regularly followed-up.

## Conclusions

Our results show a potential association between GnRH analogues therapy and the risk of PCOS development. Women treated in childhood with GnRHa due to CPP might be more likely to develop PCOS in the future. It remains unclear whether this risk is related with GnRHa treatment or with CPP itself. In this group of women, the total testosterone concentration correlated with ovarian volume. In the light of our study, strict screening for polycystic ovary syndrome seems to be obligatory in women treated with GnRH analogues in childhood. Future studies should be conducted on a large group of patients to confirm this potential relationship between GnRHa treatment and PCOS.

## Limitations

Small sample size is an important limitation of this study and it results from an extremely low incidence of CPP. Due to the limited quantity of subjects we were unable to perform more advanced statistical analysis (ie. regression analysis) to evaluate potent risk factors for PCOS. This study was single center, retrospective and not randomized. Another limitation is the control group. In an attempt to create the conditions for conducting a clinical trial, patients with a somewhat similar diagnosis were selected, with the awareness that they do not constitute the ideal representative control group.

## Data availability statement

The original contributions presented in the study are included in the article/supplementary material. Further inquiries can be directed to the corresponding author.

## Ethics statement

The studies involving humans were approved by Bioethical Committee of the Medical University of Silesia in Katowice (consent no.: KNW/0022/KBI/135/13/14). The studies were conducted in accordance with the local legislation and institutional requirements. The participants provided their written informed consent to participate in this study.

## Author contributions

DO: Conceptualization, Funding acquisition, Investigation, Methodology, Software, Writing – original draft. KN: Conceptualization, Investigation, Methodology, Visualization, Writing – original draft. AB: Data curation, Resources, Writing – original draft. AG: Supervision, Writing – review & editing. KZ: Project administration, Validation, Writing – original draft. AD-C: Formal analysis, Funding acquisition, Supervision, Validation, Writing – review & editing.
